# Prognostic Factors and Models for Changes in Cognitive Performance After Multi-Domain Cognitive Training in Healthy Older Adults: A Systematic Review

**DOI:** 10.3389/fnhum.2021.636355

**Published:** 2021-04-27

**Authors:** Mandy Roheger, Hannah Liebermann-Jordanidis, Fabian Krohm, Anne Adams, Elke Kalbe

**Affiliations:** ^1^Department of Neurology, University Medicine Greifswald, Greifswald, Germany; ^2^Department of Medical Psychology | Neuropsychology and Gender Studies and Center for Neuropsychological Diagnostics and Intervention (CeNDI), Faculty of Medicine and University Hospital Cologne, Cologne, Germany; ^3^Institute of Medical Statistics and Computational Biology, University of Cologne, Faculty of Medicine and University Hospital Cologne, Cologne, Germany

**Keywords:** prognostic factors, prognostic model, cognitive training, prediction, cognition, memory

## Abstract

**Background:** Cognitive Training (CT) may contribute to the maintenance and even enhancement of cognitive functions in healthy older adults. However, the question who benefits most from multi-domain CTs is still highly under-investigated.

**Objective:** The goal is to investigate prognostic factors and models for changes in cognitive test performance in healthy older adults after a multi-domain CT.

**Methods:** The data bases MEDLINE, Web of Science Core Collection, CENTRAL, and PsycInfo were searched up to July 2019. Studies investigating prognostic factors and/or models on cognitive outcomes (global cognition, memory, attention, executive functions, language, visuo-spatial abilities) after conducting a multi-domain CT in healthy older adults were included. Risk of Bias was assessed using the QUIPS and the PROBAST tool.

**Results:** 23 prognostic factor and model studies were included. Results indicate a high heterogeneity regarding the conducted multi-domain CTs, the investigated prognostic factors, the investigated outcomes, and the used statistical approaches. Age and neuropsychological performance at study entry were the most investigated predictors, yet they show inconsistent results.

**Conclusion:** Data on prognostic factors and models of changes after multi-domain CT are still too rare and inconsistent to draw clear conclusions due to statistical shortcomings and low reporting quality. Approaches for future research are outlined.

**Registration:** https://www.crd.york.ac.uk/prospero/, ID: CRD42020147531

## Introduction

Healthy aging is associated with various functional and structural changes in neural mechanisms leading to a decrease in cognitive functioning (Reuter-Lorenz and Park, 2014). The most vulnerable domains for age-related changes are memory, executive function, and attention (Hughes et al., 2018). Several randomized controlled trials (RCTs) provide evidence that cognitive performance of healthy older individuals can be maintained or even improved by cognitive trainings (CT) (Martin et al., 2011; Reijnders et al., 2013; e.g., Chiu et al., 2017; Shah et al., 2017). A meta-analysis suggested that CT has even long-lasting and persistent protective effects on cognition in healthy older adults (Valenzuela and Sachdev, 2009); therefore, it is increasingly taken into consideration in the prevention of age-associated neurodegenerative diseases as dementia. CTs are defined as non-pharmacological interventions using tasks focusing on memory, executive function, attention, language, and/or visuo-spatial abilities. There are many formats of CTs differing in features such as modality (paper-pencil vs. digital), setting (individual vs. group), intensity or number of trained domains (single- vs. multi-domain training). Furthermore, single-domain trainings focus on one specific cognitive function, whereas multi-domain interventions target two or more cognitive domains. Due to the stimulation of multiple cognitive processes, multi-domain CT is more closely related to real-life demands than single-domain training (Binder et al., 2015). Complementary, the multi-domain approach is frequently applied in commercially available “brain games,” such as Nintendo's Dr Kawashima's Brain Training, which are widely-used in the older population (Simons et al., 2016). Brain games aim to train cognition in a playful way by using various cognitive tasks.

Effects of multi-domain interventions could be observed in trained tasks in healthy older people; transfer effects in untrained tasks are also under discussion, although the results on this topic are heterogeneous (Walton et al., 2015). Notably, a recent meta-analysis by Basak et al. (2020) including *n* = 215 training studies found that all modules of multicomponent training yielded significant near and far transfer effects (Basak et al., 2020). Besides effects on neuropsychological measures, training-related changes have been found in EEG (Küper et al., 2017) as well as fMRI studies (Li et al., 2016) indicating plastic processes in neural functioning in the healthy aging brain. Even though the effectiveness of multi-domain CTs in healthy older adults has been described systematically in the past (Basak et al., 2020), overviews summarizing the evidence of factors and/or models predicting those training-related gains are lacking. Single studies have revealed that single variables, also called prognostic factors, as for example age (Legault et al., 2011), cognitive baseline performance (Gallen et al., 2016) or genetic markers (Bellander et al., 2015) impact the individual benefit. A prognostic factor is defined as a single factor from which risks can be calculated for a specific endpoint, whereas a prognostic or predictive model is a formal combination of multiple predictors from which risks of a specific endpoint can be calculated for individuals (Steyerberg et al., 2013). Prognostic factors for change in memory test performance after a single-domain memory training have been recently summarized by Roheger et al. (2020a). The authors emphasized methodological heterogeneity of included studies leading to inconsistent findings in prognostic factors and could show that the results vary not only as a function of the type of statistical calculation used to determine prognostic factors, but also of the type of dependent variables used in the calculations: post-test scores, change scores, relative change scores, and residual change scores. A further review on prognostic models for memory training success showed that lower age combined with higher education seems to predict higher improvements after memory training (Roheger et al., 2020b). Yet, both reviews solely focused on memory training as a specific example of a CT.

To date and to the best of our knowledge, there is no systematic review summarizing the evidence of factors and models predicting training success in multi-domain CT. Due to the interventions' broad and frequent use to prevent cognitive decline in the older population, it is in the interest of public health to elaborate who actually benefits from this treatment option. Prognostic factors such as e.g., sociodemographic, neuropsychological or neural parameters can facilitate the process of individual decision-making with regard to interventions preventing cognitive decline. Knowledge in this field would be highly relevant for decision support to realize personalized medicine. Therefore, the aim of the present study is to review specific prognostic factors and models for changes in global cognition, memory, executive function, attention, language, and visuo-spatial function (O) after multi-domain CT (I) compared to an active or passive control (C) in healthy older adults (P) in a non-clinical setting (S).

## Methods

The present systematic review was preregistered and the review protocol can be assessed at www.crd.york.ac.uk/PROSPERO/ (ID: CRD42020147531). The reporting follows the Preferred Reporting Items for Systematic Reviews and Meta-Analyses (PRISMA) guideline for systematic reviews and meta-analysis (Moher et al., 2009): “The PRISMA for Abstracts Checklists,” as well as “The PRISMA checklist for systematic reviews” are displayed in Supplementary Tables 1, 2.

### Search and Study Selection

MEDLINE (via Ovid), Web of Science Core Collection, CENTRAL and PsycInfo were systematically searched for relevant studies up to July 2019. Furthermore, reference lists of all identified trials, relevant review articles and current treatment guidelines were hand searched for further literature. In cases where no full text could be obtained, we contacted the authors and asked them to provide full text publications within a 2-week time frame. Further information on the systematic search and the full search strategies are presented in the Supplementary Tables 3–6.

Titles and abstracts were screened according to predefined eligibility criteria by two individual review authors (MR and HLJ) with the Covidence Software (https://www.covidence.org/). Full-text articles of the studies that met the inclusion criteria were further reviewed for inclusion in the systematic review. In cases where no consensus could be reached between the two authors MR and HLJ, the case was discussed until a final consensus was reached.

### Eligibility Criteria

Eligibility criteria were defined in terms of population, interventions, comparators, outcomes and timing (PICOT). The review focused on peer-reviewed studies in English and German which investigated prognostic factors and models of changes in cognitive test performance after multi-domain CT with no limitations regarding publication date. Full study reports needed to be available; abstracts, books, book chapters, study protocols, and conference papers were excluded.

Studies including data on prognostic factors and models for changes after multi-domain CT performance on healthy older participants (age ≥ 55 years) were included (P). We excluded data from participants with diagnosis of cognitive impairment or dementia, neurological and/or psychiatric diseases, assessed at least via self-report.

Regarding the included prognostic factors and models, all prognostic factors (e.g., sociodemographic factors, brain imaging parameters, genetic parameters, blood factors, personality traits, cognitive abilities at the entry of the training, different training characteristics, e.g., intensity of the trainings, etc.) and all prognostic models which investigate changes in cognitive test performance after multi-domain CT were included in the review and meta-analysis. Multi-domain CT was defined as a CT that includes tasks for training of at least two cognitive domains. The training should consist of at least 90% of cognitive exercises (next to e.g., physical exercises, life-style interventions, diets) with a minimum of two sessions in total. Cognitive domains could either be trained separately and sequentially, or several cognitive domains could be trained simultaneously. Furthermore, the training could either include computerized or paper-pencil tasks with clear cognitive rationale, which were administered either on personal devices or in individual- or group settings (I). No pre-assumptions about comparator interventions were made (C). Regarding videogame trainings and brain trainings, we only included those in which cognitive domains trained were explicitly outlined.

Studies including data on prognostic factors and models, which investigate cognitive changes after training as an outcome (global cognition, memory, attention, executive functions, language, visuo-spatial abilities) measured with established objective neuropsychological tests, were included (O). The factor measurement of the included studies had to be conducted before the training started, and there was no limitation regarding post-measurements of outcomes or the length of the follow-ups (T).

### Data Extraction

Two review authors (MR and HLJ) independently extracted the data according to the Critical appraisal and data extraction for systematic reviews of prediction modeling studies_ prognostic factors (CHARMS_PF) checklist (Moons et al., 2014) to investigate the reporting of prognostic factors.

### Quality Assessment

Two reviewers (MR and HLJ) independently assessed the risk of bias of included studies. For prognostic factor studies, the Quality in Prognosis Studies (QUIPS) checklist, developed by Hayden and colleagues (2013) was used to examine the risk of bias in prognostic factor studies across six domains (Hayden et al., 2013): Study participation, study attrition, prognostic factor measurement, outcome measurement, adjustment for other prognostic factors, statistical analyses, and reporting. Each of the six domains was judged with high, moderate or low risk. A detailed description of the domains included in the tool and the judgement taken by the two reviewers are presented in Supplementary Table 7.

Prognostic model studies were assessed using the “Prediction model Risk of Bias Assessment Tool (PROBAST)” (Wolff et al., 2019) which examines the risk of bias in prognostic model studies across four domains: Participants, Predictors, Outcome, Analysis. Every domain was voted with “yes,” “probably yes,” “no,” “probably no,” and “no information” to rate their risk of bias. To ensure fairness in the ratings, risk of bias assessment of a study conducted by two of the authors of the present review [MR, EK] was done independently by two researchers [HLJ, FK] who were not involved in the former study (Roheger et al., 2020c).

### Statistical Analysis

The extracted data was entered into an electronic database by a review author [MR] and checked by a second review author [HLJ]. All analyses should be conducted with the statistic program R version 3.5.0. Predictors and models of included studies should be examined using meta-analyses (separately for factors and models).

In the preregistration of the present study, it was planned that if clinical and methodological characteristics of the individual studies were sufficiently homogeneous, statistical measures for model performance (e.g., statistics for discrimination and calibration) and model parameters (e.g., regression coefficients) should be pooled meta-analytically across studies and a weighted mean including the corresponding 95% confidence interval should be calculated. Additionally, forest plots were planned to graphically present the results. Multivariable models could only be pooled if the same or at least a very similar set of prognostic factors were used to adapt the model. Random-effects models should be used for meta-analyses.

To evaluate the presence of heterogeneity between studies, heterogeneity statistics *I*^2^ und tau^2^ should be calculated and examined using a chi-squared test (Q-test). Interpretation of the *I*^2^ statistic should be made according to chapter 9.5.2 of the Cochrane Handbook for Systematic Reviews of Interventions (Higgins, 2008). Relevant heterogeneity should be also examined using meta-regression and subgroup analyses.

However, after data extraction, we found that data on prognostic factors and models of changes after CT were too heterogeneous and too poorly reported to conduct a meta-analysis.

## Results

### Study Selection

**Figure 1:**
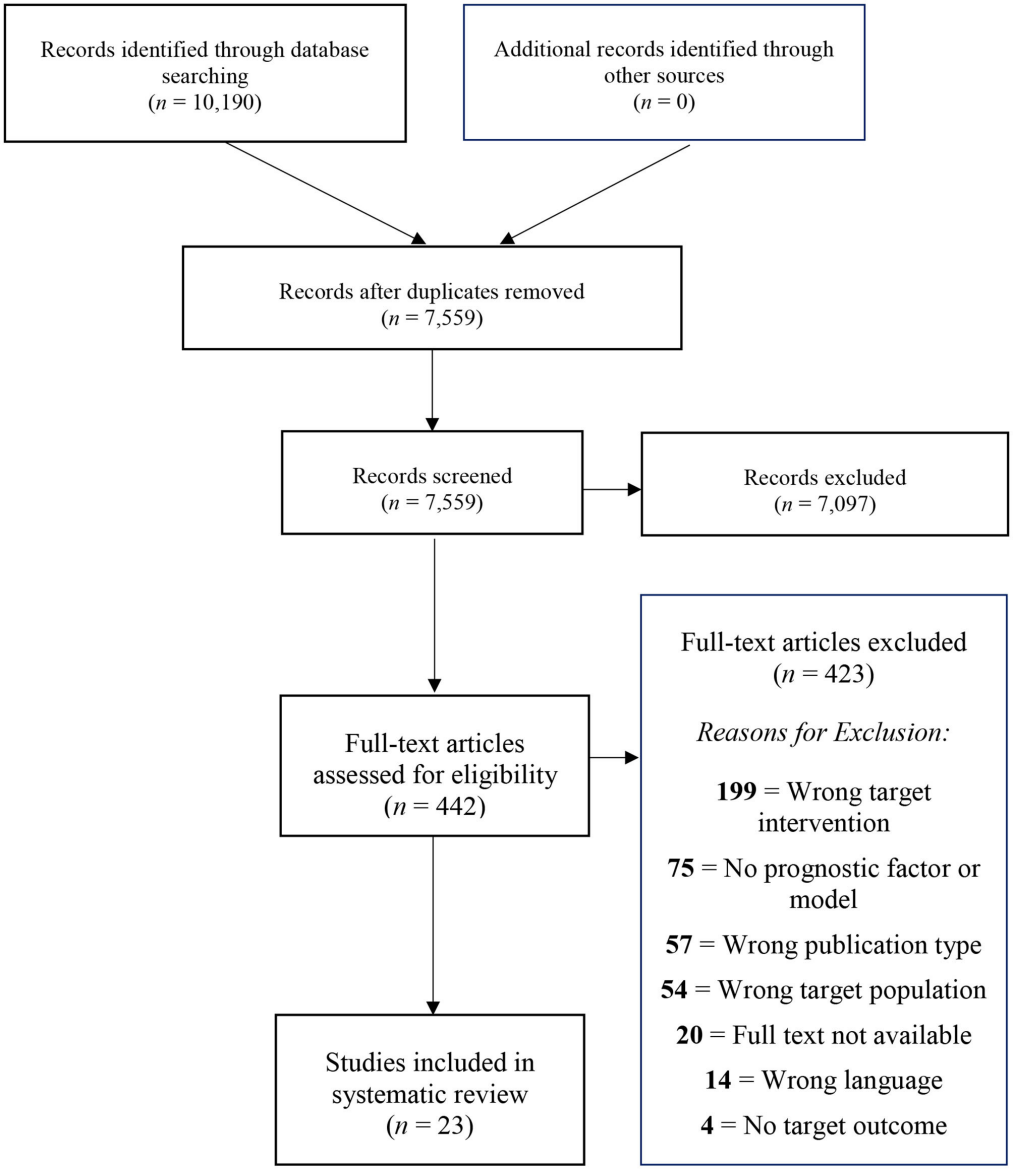
PRISMA diagram showing the study selection process.

The total number of retrieved references and the numbers of included and excluded studies with reasons for exclusion are documented in a flow chart as recommended in the PRISMA statement (Moher et al., 2009; Figure 1). In total, *n* = 10,190 studies were identified through the database search. After removing the duplicates, *n* = 7,559 studies were screened. We assessed *n* = 446 full-texts for eligibility. Finally, *n* = 23 studies were included in the present review. All included studies were published in English.

### Study and Participants' Characteristics

An overview of the main characteristics of the included studies [i.e., study type (Prognostic factor vs. prognostic model study), initial sample sizes of the multi-domain CT groups, sample characteristics (age, sex, and education), detailed description of CT] is outlined in Table 1.

**Table 1:** Participants' demographics and memory training characteristics.

**Study**	**Study type**		**Participants**					**Training**							
**Author** **(year)**	**Prognostic** **model**	**Prognostic** **factor**	**n^a^**	**Age** **(in years)** **M (SD)**	**Sex**	**Education** **(in years) M (SD)**	**Global Cognition** **(at baseline)** **Test M (SD)**	**Total Time** **(in minutes)**	**Frequency**	**Description of** **training**	**Psychoeducation?**	**Group setting?**	**Digital?**	**Strategy?**	**Specific tasks?**
Akimoto et al. (2016)		x	Group P: 9 Group V: 5	Group P: 68.71 (5.96) Group V: 67.34 (5.59)	Group P: 4 ♀, 5 ♂ Group V: 1 ♀, 4 ♂	Group P: 13.44 (2.40) Group V: 13.60 (2.19)	MMSE: Group P: 28.00 (1.80) Group V: 28.00 (1.23)	480	24 sessions, 3 days a week for 8 weeks	Cognitive intervention: Group V: vehicle training Group P: Personal computer training			x		x
Anderson et al. (2014)		x	30	62.30 (3.40)	16 ♀, 14 ♂	n.a.	WAIS: 119 (11)	2,400	40 sessions, 5 sessions per week for 8 weeks	Auditory-based CT, Brain Fitness program			x		x
Bellander et al. (2015)	x		103	71.30 (4.10)	51 ♀, 52 ♂	13.60 (3.60)	n.a.	6,060	101 days	CT with 12 computerized tasks			x		x
Binder et al. (2016)	x		21	69.62 (2.85)	13 ♀, 8 ♂	9.55 (1.61)	MMSE: 28.90 (0.89)	Max. 3,000	50 sessions, 5 sessions per week for 10 weeks	Multi-domain CT			x		x
Fernández-Prado et al. (2012)		x	n.a.	n.a.	n.a.	n.a.	n.a.	n.a.	n.a.	Cognitive stimulation program		x			x
Gallen et al. (2016)		x	24	63.10 (2.90)	9 ♀, 15 ♂	n.a.	IQ: 122.1 (8.3)	2,160	1 group session per week over 12 weeks, 2 individual sessions per week over 12 weeks	CT (SMART)		x		x	
Kim et al. (2015)		x	Traditional: 24 Robot: 24	Traditional: 67.70 (5.40) Robot: 68.00 (6.10)	Traditional: 6 ♀, 18 ♂ Robot: 10 ♀, 14 ♂	Traditional: 14.00 (3.30) Robot: 13.20 (3.90)	K-MMSE Traditional: 29.10 (0.90) Robot: 28.90 (1.50)	5,400	60 sessions, 5 days per week over 12 weeks	Traditional and robot-assisted multi-domain CT		x	x		x
Legault et al. (2011)		x	18	76.00 (5.20)	8 ♀, 10 ♂	n.a.	MMMSE: 95.60 (3.40)	1,152	24 sessions, 8 per month during Months 1-2 and 4 per month during Months 3-4	CT (Active Mind)		x	x		x
López-Higes et al. (2018a)	x		CI:32 SCD:49	CI: 70.94 (4.16) SCD: 71.41 (4.83)	CI: 20 ♀, 12 ♂ SCD: 35 ♀, 14 ♂	CI: 14.38 (5.88) SCD: 13.13 (5.96)	MMSE: CI:28.94 (1.19) SCD:28.35 (1.63)	2,700	3 sessions per week over 13 weeks	UMAM CT Program		x			x
López-Higes et al. (2018b)	x		66, CI: 31 SCD: 35	CI: 70.94 (4.16); SCD: 71.39 (4.96)	CI: 12 ♂, 19 ♀; SCD: 10 ♂, 25 ♀	CI: 14.38 (5.88); SCD: 13.02 (6.05)	MMSE: CI: 28.94 (1.19); SCD: 28.45 (1.50)	2,700	3 sessions per week over 13 weeks	UMAM CT Program		x			x
Lövdén et al. (2010)		x	12	68.90 (2.70)	5 ♂, 7 ♀	n.a.	n.a.	6,060	Up to 101 sessions	CT with 12 computerized tasks			x		x
Mayas et al. (2014)		x	15	68.70 (5.20)	6 ♂, 9 ♀	11.90 (4.80)	MMSE: 28.9 (1.00)	1m200	20 1h sessions across a period of 10-12 weeks	Video training Lumosity			x		x
McDougall and House (2012)		x	21	74.81 (7.85)	11 ♂, 10 ♀	n.a.	n.a.	n.a.	6 weeks, participants should use it regularly	Nintendo “Brain Training”			x		x
Miller et al. (2013)	x		42	82.20 (4.40)	15 ♂, 27 ♀	16.20 (2.20)	MMSE: 28.00 (1.50); MocA: 24.20 (3.10)	800	5 days a week for 20–25 min each day for 8 weeks	Brain fitness			x		x
Niu et al. (2016)		X	16	69.60 (4.60)	7 ♂, 9 ♀	12.40 (3.50)	MMSE: 27.70 (1.50)	960	16 sessions over 6 weeks	Combined CT				x	x
Nouchi et al. (2019)	x		27	71.67 (3.62)	n.a.	12.96 (2.01)	MMSE: 28.93 (1.14)	Minimum: 600	at least 5 days per week, for a total of 6 weeks	CT game for car driving group			x		x
Oswald et al. (1996)		x	272 in total	n.a.	n.a.	n.a.	n.a.	Minimum: 3,600	Once a week over 30 sessions	Competence-training Program		x			x
Otsuka et al. (2015)		X	14	82.21 (2.89)	n.a.	11.07 (2.53)	MMSE: 27.50 (2.14)	1,125	Once a week for 4 times a month, for about 6 months	Atama-nodojo		x	x		x
Polito et al. (2015)	x		38	73.80 (1.20)	17 ♂, 21 ♀	7.70 (3.00)	MMSE: 28.20 (1.50), Moca: 23.20 (3.60)	1,000	10 twice weekly sessions	Cognitive stimulation	x	x		x	
Roheger et al. (2020c)	x		ST: 20 LT: 17	ST: 67.65 (6.86) LT: 68.35 (6.01)	ST: 12 ♀ 8♂ LT: 7 ♂, 10 ♀	ST:14.80 (2.82) LT:14.53 (2.90)	DemTecT: ST: 16.60 (1.70) LT:16.76 (1.56)	1,260	2 sessions over 7 weeks	NeuroVitalis CT	x	x			x
Schmiedek et al. (2010)	x		101	71.30 (4.10)	50 ♂, 51 ♀	13.60 (3.60)	n.a.	6,060	Up to 101 sessions	CT with 12 computerized tasks			x		x
Shah et al. (2014)		x	51	66.61 (4.82)	19 ♂, 32 ♀	11.00 (2.00)	MMSE: 28.80 (1.18)	2,400	40 sessions over 16 weeks	Cognitive Stimulation; auditory-based Brain Fitness Program (BFP) and the visual-based Insight Program (IP)			x		x
Shing et al. (2012)	x		101	71.30 (4.10)	50 ♂, 51 ♀	13.60 (3.60)	n.a.	6,060	Up to 101 sessions	CT with 12 computerized tasks			x		x

*MMSE, Mini Mental State Examination; MMMSE, Modified Mini Mental State Examination; K-MMSE, Korean version of the Mini Mental Status Examination; WAIS, Wechsler Adult Intelligence Scale; CI, Cognitively Intact, SCD, Subjective Cognitive Decline; IQ, Intelligence Quotient; MoCA, Montreal Cognitive Assessment; ST, short-term, LT, long-term; DemTect, Demenz-Detektionstest.*

a *Total number of participants in CT group of older participants*.

In total, *n* = 13 of the included studies investigated prognostic factors, whereas the other *n* = 10 studies investigated prognostic models to measure changes in cognitive functions after multi-domain CT. The sample sizes of the CT groups varied throughout the prognostic factor studies between *n* = 5 participants (Akimoto et al., 2016) and *n* = 272 participants (Oswald et al., 1996, even though no further information on participants is provided in this study report) and between *n* = 21 participants (Binder et al., 2016) and *n* = 103 participants (Schmiedek et al., 2010; Shing et al., 2012; Bellander et al., 2015) in the prognostic model studies. Participants were between 62.3 years (Anderson et al., 2014) and 82.21 years old (Otsuka et al., 2015) in the prognostic factor studies, and between 67.65 (Roheger et al., 2020c) and 82.20 years old (Miller et al., 2013) in the prognostic model studies. In both the prognostic factor and prognostic model studies, an almost equal number of male and female participants was investigated (overall: 51% male and 49% female participants; 46% male and 54% female participants, respectively). Three prognostic studies (Oswald et al., 1996; Fernández-Prado et al., 2012; Otsuka et al., 2015) and one prognostic model study (Nouchi et al., 2019) did not report sex distribution among the participants. Years of education varied in the prognostic factor studies between 11.00 (Shah et al., 2014) and 14.00 (Kim et al., 2015), and between 7.70 (Polito et al., 2015) and 14.80 (Roheger et al., 2020c) in the prognostic model studies. *N* = 7 studies did not report education at all. Global cognitive status was assessed in *n* = 16 studies with heterogeneous tests. The Mini-Mental Status Examination (MMSE) was assessed in most of them with *n* = 13.

### Multi-Domain Cognitive Training Characteristics

Characteristics of the multi-domain CTs used in the included studies are depicted in Table 1. It has to be emphasize that it was difficult to determine which specific cognitive domains were trained in the CTs, as often only the used tasks were described without a clear classification to the corresponding domains. Therefore, the review authors classified tasks and tests to their best knowledge. Overall, there was a huge heterogeneity not only in the total time of training in minutes [varying from 480 min (Akimoto et al., 2016) to 6,060 min (Lövdén et al., 2010; Schmiedek et al., 2010; Shing et al., 2012; Bellander et al., 2015)], but also regarding frequency, number of trained domains, and content of the trainings.

CTs were clustered regarding the fact whether they included psychoeducation, were held in individual or group settings, were digital or in paper-pencil-form or whether they included learning of strategies or focused more on specific training tasks. *N* = 15 studies used a digital CT with tasks training specific functions [prognostic factor studies: *n* = 9 (Oswald et al., 1996; Lövdén et al., 2010; Legault et al., 2011; McDougall and House, 2012; Anderson et al., 2014; Mayas et al., 2014; Shah et al., 2014; Kim et al., 2015; Akimoto et al., 2016), prognostic model studies: *n* = 6 (Schmiedek et al., 2010; Shing et al., 2012; Miller et al., 2013; Bellander et al., 2015; Binder et al., 2016; Nouchi et al., 2019)]. A total of *n* = 10 studies were held in group settings [*n* = 6 of them prognostic factor studies (Oswald et al., 1996; Legault et al., 2011; Fernández-Prado et al., 2012; Kim et al., 2015; Otsuka et al., 2015; Gallen et al., 2016), *n* = 4 prognostic model studies (Polito et al., 2015; López-Higes et al., 2018a,b; Roheger et al., 2020c)].

Greyly marked cognitive domains in Table 2 (prognostic factor studies) and Table 3 (prognostic model studies) symbolize the specific domains the participants were trained in the cognitive multi-domain training.

**Table 2:** Detailed results of prognostic factor studies.

**Study**	**Prognostic factor**	**Dependent variable**	**Outcomes and tests**					
			**Global cognition**	**Memory**	**Attention**	**Executive function**	**Language**	**Visuo-spatial abilities**
**Correlation analysis**								
Akimoto et al. (2016)	High gamma power change	Change score (Post-Pre)	x			x		
Anderson et al. (2014)	Peak variability	Change score (Post-Pre)		↓		↓^*^ processing speed measured with WJIII		
Fernández-Prado et al. (2012)	Subjective health (measured with CUBRECAVI)	n.a.	↑^*^ measured with MEC					
Gallen et al. (2016)	1. Baseline 2. Baseline whole-brain modularity 3. Modularity of sub-networks 4. whole Brain Network Network Segregation	Change score (Post-Pre)				1. ↓^*^ measured with TOSL 2. ↑^*^ measured with TOSL 3. ↑^*^ measured with TOSL 4. ↑ measured with TOSL		
Kim et al. (2015)	Cortical thickness in the right inferior temporal gyrus and right subgenual cingulate region	Change score (Post-Pre)	x	↑^*^ measured with PRM task		x		
Legault et al. (2011)	1. Age 2. Education 3. ApoE4	Composite change scores (Post-Pre)	1. ↓ All measured with composite score 2. x 3. x	1. ↓ All measured with composite score 2. x 3. x		1. ↓ All measured with composite score 2. x 3. x		
Lövdén et al. (2010)	1. Mean diffusivity 2. Fractional anisotropy	Composite change scores (Post-Pre)		x		x		
Mayas et al. (2014)	1. Alertness 2. Distraction	Change score (Post-Pre)				1. ↓^*^ Measured with speed game 2. ↑^*^ Measured with “lost in migration migration game		
McDougall and House (2012)	1. Perceived cognitive function 2. Quality of life	Standardized change score (Post-Pre)				1. ↓^*^ Measured with digit span 2. ↑^*^ Measured with digit span	1. ↓ Measured with vocabulary test 2. ↑^*^ Measured with vocabulary test	
Niu et al. (2016)	1. RP amplitude 2. CNV amplitude	Standardized change score (Post-Pre)		1. x 2. x		1. ↑^*^ Measured with picture updating 2. ↑^*^ Measured with picture updating		
Oswald et al. (1996)	1. Age 2. Sex 3. Baseline performance	Standardized change score (Post-Pre)	1. x 2. x 3. ↓^*^	1. x 2. x 3. ↓^*^		1. x 2. x 3. ↓^*^		
Otsuka et al. (2015)	1. Age 2. Education 3. Attendance to the session 4. Depression measured with GDS	Change score (Post-Pre)	1. x 2. x 3. x 4. ↓ measured with FAB	1. x 2. x 3. x 4. x	1. x 2. x 3. x 4. x			
Shah et al. (2014)	1. SMC-L 2. FRT-L	n.a.		1. ↓ 2. ↓ measured with long Term Delayed Recall			1. ↑ 2. ↓ Measured with COWAT	

*WJIII, Woodcock–Johnson III Tests of Cognitive Abilities Visual Matching sub-test; MEC, Lobo's Cognitive Mini-Exam, CUBRECAVI, Short Questionnaire on Quality of Life; TOSL, Test of Strategic Learning; PRM, Pattern Recognition Memory; ApoE4, Apoliopoprotein E 4; GDS, Geriatric Depression Scale; FAB, Frontal Assessment Battery; SMC-L, left sensorimotor cortex; FRT-L, left frontal cortex; COWAT, Controlled oral word association test; Gray Outcome domains symbolize the domains the participants were trained in the CT. x, no direction of relationship between prognostic factor and outcome was indicated, ↑ = a positive relationship was reported between the prognostic factor and the outcome, ↓ = a negative relationship was reported between the prognostic factor and the outcome*,

* *a significant relationship (p < 0.05) was reported*.

**Table 3:** Detailed results of prognostic model studies.

**Study**	**Prognostic factors**	**Dependent variables**	**Outcomes and used tests**					
			**Global cognition**	**Memory**	**Attention**	**Executive function**	**Language**	**Visuo-spatial abilities**
**Latent change score model**								
Bellander et al. (2015)	1. LMX1A 2. DRD2 3. COMT	Change score (Post-Pre)		1. x 2. x 3. x		1. x 2. x 3. ↑^*^ working memory		
Schmiedek et al. (2010)	Age	Net effect scores		x		x		
Shing et al. (2012)	Age	Change score (Post-Pre)		↓		x		
**Structural equation model**								
Binder et al. (2016)	Baseline performance	Composite latent change score				↓^*^		x
**Linear regression model**								
López-Higes et al. (2018a)	Cognitive reserve measured with digit reordering baseline performance	Change score (Post-Pre)	↓^*^ measured with MMSE					
López-Higes et al. (2018b)	Cognitive reserve measured with interference baseline performance	Change score (Post-Pre)					↓^*^ measured with visual confronting naming	
Miller et al. (2013)	Number of sessions (Dose)	Standardized composite change score (Post-Pre)		x			x	
Nouchi et al. (2019)	1. Age 2. Sex 3. MMSE 4. Baseline Performance	Change Score (Post-Pre)		1. x 2. x 3. x 4. x	1. x 2. x 3. x 4. x	1. x 2. x 3. x 4. ↑^*^measured with symbol coding		
Polito et al. (2015)	1. ApoE4: Carrier 2. Sex: Female	Net change score	1. ↓ measured with MMSE and MoCa 2. ↓ measured with MMSE and MoCa					
Roheger et al. (2020c)	1. Age 2. Sex: Female 3. Education 4. Baseline 5. ApoE4: Carrier 6. IGF-1 7. VEGF 8. BDNF	Change scores (Post-Pre)	1. x 2. x 3. x 4. ↓^*^ measured with DemTect 5. x 6. x	1. x 2. ↓^*^ measured with ROFDR 3. x 4. ↓^*^ measured with word list 5. x 6. x	1. x 2. x 3. x 4. ↓^*^ measured with BTA 5. x 6. x	1. x 2. ↓^*^ measured with key search 3. x 4. ↓^*^ measured with TMT A/B 5. x 6. x	1. x 2. x 3. ↓^*^ measured with letter fluency 4. ↓^*^ measured with letter fluency 5. x 6. x	1. x 2. x 3. x 4. x 5. ↓^*^ measured with ROF 6. x

*MMSE, Mini Mental Status Examination; ApoE4, Apolipoprotein 4; MoCa, Montreal Cognitive Assessment; DemTect, Demenz-Detektionstest; ROFDR, Rey Osterrieth Figure: delayed recall; BTA, Brief Test of Attention; TMT A/B, Trial Making Test A/B; IGF-1, Insulin-like growth factor 1; VEGF, Vascular Endothelial Growth Factor; BDNF, Brain-derived neurotrophic factor; Gray Outcome domains symbolize the domains the participants were trained in the CT. x = no direction of relationship between prognostic factor in the model and outcome was indicated, ↑ = a positive relationship was reported between the prognostic factor in the model and the outcome, ↓ = a negative relationship was reported between the prognostic factor in the model and the outcome*,

* *a significant relationship (p < 0.05) was reported*.

In the prognostic factor studies, all except two CTs (Fernández-Prado et al., 2012; Gallen et al., 2016) mainly focused on executive functions. Memory was the second most trained cognitive domain in the prognostic factor studies in nine out of 13 studies (Oswald et al., 1996; Lövdén et al., 2010; Legault et al., 2011; Fernández-Prado et al., 2012; Shah et al., 2014; Kim et al., 2015; Otsuka et al., 2015; Gallen et al., 2016; Niu et al., 2016). *N* = 5 studies trained attention (Fernández-Prado et al., 2012; Anderson et al., 2014; Otsuka et al., 2015; Akimoto et al., 2016; Gallen et al., 2016), *n* = 4 language (Fernández-Prado et al., 2012; McDougall and House, 2012; Kim et al., 2015; Otsuka et al., 2015), *n* = 3 global cognition (Fernández-Prado et al., 2012; Mayas et al., 2014; Otsuka et al., 2015), and *n* = 2 visuo-spatial abilities (McDougall and House, 2012; Kim et al., 2015). None of the studies offered a CT that trained all cognitive domains.

In the prognostic model studies, all studies trained the domain executive functions. Memory was trained in *n* = 8 studies (Lövdén et al., 2010; Schmiedek et al., 2010; Shing et al., 2012; Miller et al., 2013; Bellander et al., 2015; Polito et al., 2015; López-Higes et al., 2018b; Roheger et al., 2020c), attention in *n* = 5 (Polito et al., 2015; López-Higes et al., 2018a,b; Nouchi et al., 2019; Roheger et al., 2020c), language in *n* = 4 (Miller et al., 2013; Polito et al., 2015; López-Higes et al., 2018a,b), global cognition in *n* = 3 (Polito et al., 2015; López-Higes et al., 2018a,b), and visuo-spatial abilities in *n* = 3 (Miller et al., 2013; Polito et al., 2015; Binder et al., 2016). Only one prognostic model study included a CT targeting all cognitive domains (Polito et al., 2015).

### Risk of Bias

Results of the Risk of Bias Assessment are displayed in Tables 4 and 5. Risk of Bias assessment for prognostic factor studies showed a lack of reporting in the domains “Study Attrition” and “Study Confounders” in most of the studies, probably as a result of the fact that often prognostic factor assessment was not the main goal of the included studies, but more an “add-on”. Overall, prognostic model studies showed a medium reporting quality, yet, several studies were lacking information in the domain “statistical analyses” and especially on model validation.

**Table 4:** Risk of bias assessment for prognostic factor studies.

	**Study participation**	**Study attrition**	**Prognostic factor measurement**	**Outcome measurement**	**Study confounding**	**Statistical analysis and reporting**
Akimoto et al. (2016)						
Anderson et al. (2014)						
Fernández-Prado et al. (2012)						
Gallen et al. (2016)						
Kim et al. (2015)						
Legault et al. (2011)						
Lövdén et al. (2010)						
Mayas et al. (2014)						
McDougall and House (2012)						
Niu et al. (2016)						
Oswald et al. (1996)^a^						
Otsuka et al. (2015)						
Shah et al. (2014)						

*Red color indicates a high risk of bias, yellow color indicates a moderate risk of bias, green color indicates a low risk of bias, assessed with the QUIPS tool (Hayden et al., 2013)*.

a *Note that even though the quality rating for the study of Oswald et al. (1996) was quite low, the author was the only one who provided additional data and study information when asked by the review authors*.

**Table 5:** Risk of bias assessment for prognostic model studies.

	**Bellander et al. (2015)**	**Binder et al. (2016)**	**López-Higes et al. (2018a)**	**López-Higes et al. (2018b)**	**Miller et al. (2013)**	**Nouchi et al. (2019)**	**Polito et al. (2015)**	**Roheger et al. (2020c)**	**Schmiedek et al. (2010)**	**Shing et al. (2012)**
**Participants**										
Where appropriate data sources used, e.g. cohort, RCT?										
Were all inclusions and exclusions of participants appropriate?										
**Predictors**										
Were predictors defined and assessed in a similar way for all participants?										
Were predictor assessments made without knowledge of outcome data?										
Are all predictors available at the time the model is intended to be used?										
**Outcome**										
Was the outcome determined appropriately?										
Was a pre-specified or standard outcome definition used?										
Were the predictors excluded from the outcome definition?										
Was the outcome defined in a similar way for all participants?										
Was the outcome defined without knowledge of predictors?										
Time interval between outcome and predictor appropriate?										
Were there a reasonable number of participants with the outcome?										
**Analysis**										
Were continuous and categorical predictors handled appropriately?										
Were all enrolled participants included in the analysis?										
Were missing data handled appropriately?										
Was selection of predictors based on univariable analysis avoided?										
Were complexities in the data accounted for (e.g., censoring, control participants?)										
Were relevant model performance measures evaluated?										
Were over- and under-fitting accounted for?										
Do predictors and weights correspond to the results from multivariable analysis?										

*Risk of bias assessment using the “Prediction model Risk of Bias Assessment Tool (PROBAST)” (Wolff et al., 2019) to examine the risk of bias in prognostic factors studies across four domains: Participants, Predictors, Outcome, Analysis. Each of the six domains was judged with “yes” (depicted in dark green), “probably yes” (light green), “no” (dark red), “probably no” (light red), and “no information” (yellow)*.

### Outcomes of Multi-Domain CT Studies

In the present review, we investigated six outcomes: global cognition, memory, attention, executive function, language, visuo-spatial abilities. Outcomes were well-defined in all investigated studies. An overview of the cognitive outcomes investigated in both, prognostic factor and prognostic model studies, can be obtained from Table 6.

**Table 6:** Overview of prognostic factors/models and cognitive outcomes of the reviewed studies.

**Study**	**Prognostic factors**									**Outcomes**					
	**Baseline** **performance**	**Age**	**Sex**	**Education**	**Psychological** **variables**	**Training** **variables**	**Genetics**	**Imaging** **marker**	**EEG** **marker**	**Global** **cognition**	**Memory**	**Attention**	**Executive** **function**	**Language**	**Visuo-spatial** **abilities**
**Prognostic factors studies**															
Akimoto et al. (2016)								x High gamma power change		x			x		
Anderson et al. (2014)									x Peak variability		x		x		
Fernández-Prado et al. (2012)					x QoL					x					
Gallen et al. (2016)	x							x Baseline whole-brain modularity, modularity of sub-networks, whole brain network segregation					x		
Kim et al. (2015)								Cortical thickness		x	x		x		
Legault et al. (2011)		x		x			x			x	x		x		
Lövdén et al. (2010)								x Mean diffusivity, fractional anisotropy			x		x		
Mayas et al. (2014)					x Alertness, distraction								x		
McDougall and House (2012)					x QoL, Perceived cognitive functioning						x		x	x	
Niu et al. (2016)									x CNV amplitude, Readiness potential, P3		x		x		
Oswald et al. (1996)	x	x	x							x	x		x		
Otsuka et al. (2015)		x		x	x Depression	x Attendance, impression				x	x	x			
Shah et al. (2014)								x Regional Counts in left sensorimotor Cortex and left frontal cortex			x			x	
**Prognostic model studies**															
Bellander et al. (2015)							x						x		
Binder et al. (2016)	x											x	x		
López-Higes et al. (2018a)	x				x Cognitive reserve					x					
López-Higes et al. (2018b)	x				x Cognitive reserve					x					
Miller et al. (2013)						x Training dose					x			x	
Nouchi et al. (2019)	x	x	x								x	x	x		
Polito et al. (2015)			x				x			x	x				
Roheger et al. (2020c)	x	x	x	x			x			x	x	x	x		x
Schmiedek et al. (2010)		x									x		x		
Shing et al. (2012)		x				x Practice							x		

*ApoE4, Apolipoprotein E4; QoL, Quality of Life; CNV, Contingent Negativity Variation*.

Executive functions was the outcome that was assessed in most studies [*n* = 10 in prognostic factor studies (Oswald et al., 1996; Lövdén et al., 2010; Legault et al., 2011; McDougall and House, 2012; Anderson et al., 2014; Mayas et al., 2014; Kim et al., 2015; Akimoto et al., 2016; Gallen et al., 2016; Niu et al., 2016), *n* = 6 in prognostic model studies (Schmiedek et al., 2010; Shing et al., 2012; Bellander et al., 2015; Binder et al., 2016; Nouchi et al., 2019; Roheger et al., 2020c)], followed by memory [*n* = 9 in prognostic factor studies (Oswald et al., 1996; Lövdén et al., 2010; Legault et al., 2011; McDougall and House, 2012; Anderson et al., 2014; Shah et al., 2014; Kim et al., 2015; Otsuka et al., 2015; Niu et al., 2016), *n* = 5 in prognostic model studies (Schmiedek et al., 2010; Miller et al., 2013; Polito et al., 2015; Nouchi et al., 2019; Roheger et al., 2020c)]. Global cognition was assessed in *n* = 6 prognostic factor studies (Oswald et al., 1996; Legault et al., 2011; Fernández-Prado et al., 2012; Kim et al., 2015; Otsuka et al., 2015; Akimoto et al., 2016), but only in two prognostic model studies (Polito et al., 2015; Roheger et al., 2020c), whereas attention was assessed in *n* = 3 prognostic model studies (Binder et al., 2016; Nouchi et al., 2019; Roheger et al., 2020c), but only in one prognostic factor study (Otsuka et al., 2015). Language and visuo-spatial abilities were the least assessed outcomes in the studies; language was only assessed in two prognostic factor studies (McDougall and House, 2012; Shah et al., 2014) and in one prognostic model study (Miller et al., 2013), visuo-spatial abilities were assessed in none of the prognostic factor studies and only in one of the prognostic model studies as an outcome (Roheger et al., 2020c). None of the studies investigated all cognitive outcome domains.

### Prognostic Factors and Models: Statistical Analyses

No detailed description [e.g., a separate paragraph stating not only the name of the predictor and method of measurement, but also blinding, and use in the statistical analysis (e.g. as a continuous or dichotomous factor)] of the candidate predictors was provided in most of the prognostic factor studies, probably as a consequence of the fact that the prediction analysis was mostly not the primary goal of the investigated studies. In prognostic model studies, the descriptions of the statistical analyses were far more detailed.

Investigated predictors include sociodemographic variables (i.e,. age, sex, and education), neuropsychological test status at study entry in different domains, further psychological variables (i.e., quality of life, depression), training characteristics (i.e., modality, intensity), genetic variables (i.e., apolipoprotein E4), brain imaging measures, and EEG markers (for an overview see Table 6, for more details see Table 2 for prognostic factor studies and Table 3 for prognostic model studies).

Prognostic factors in prognostic factor studies were highly heterogeneous. *N* = 5 studies assessed brain imaging marker (Lövdén et al., 2010; Shah et al., 2014; Kim et al., 2015; Akimoto et al., 2016; Gallen et al., 2016), however, these markers were different over all the studies. Age was assessed in *n* = 3 prognostic factor studies (Oswald et al., 1996; Legault et al., 2011; Otsuka et al., 2015), cognitive baseline performance (Oswald et al., 1996; Gallen et al., 2016) and education (Legault et al., 2011; Otsuka et al., 2015) were each assessed in *n* = 2 studies. Sex (Oswald et al., 1996), training variables (Otsuka et al., 2015), and apolipoprotein E4 genotyping (Legault et al., 2011) were each assessed in only one study. Psychological variables were used as predictors in *n* = 4 studies (Fernández-Prado et al., 2012; McDougall and House, 2012; Mayas et al., 2014; Otsuka et al., 2015), two of them assessing Quality of life (Fernández-Prado et al., 2012; McDougall and House, 2012), one assessing distraction and alertness (Mayas et al., 2014), and one assessing mood (Otsuka et al., 2015). EEG markers were used in two studies as predictors for changes after multi-domain CT (Anderson et al., 2014; Niu et al., 2016).

In all prognostic factor studies, only correlational analyses were conducted. The dependent variables were the raw change score in *n* = 6 studies (Anderson et al., 2014; Mayas et al., 2014; Kim et al., 2015; Otsuka et al., 2015; Akimoto et al., 2016; Gallen et al., 2016), the standardized change score in *n* = 3 studies (Oswald et al., 1996; McDougall and House, 2012; Niu et al., 2016), and the composite change score in *n* = 2 studies (Lövdén et al., 2010; Legault et al., 2011), while *n* = 2 studies did not clearly report their dependent variable (Fernández-Prado et al., 2012; Shah et al., 2014).

In the prognostic model studies, *n* = 5 assessed baseline performance of the investigated outcome as a predictor (Binder et al., 2016; López-Higes et al., 2018a,b; Nouchi et al., 2019; Roheger et al., 2020c). *N* = 4 studies investigated age (Schmiedek et al., 2010; Shing et al., 2012; Nouchi et al., 2019; Roheger et al., 2020c), *n* = 3 studies investigated sex as possible predictor in the model (Polito et al., 2015; Nouchi et al., 2019; Roheger et al., 2020c). Only one study assessed education (Roheger et al., 2020c), whereas two studies assessed psychological variables (López-Higes et al., 2018a,b), namely cognitive reserve, and two studies integrated training variables (attendance to training, training dose) in their model (Shing et al., 2012; Miller et al., 2013). Apolipoprotein E4, a protein involved in the metabolism of fats in the body and a risk factor for developing Alzheimer's disease, was integrated as prognostic factor in the model in *n* = 3 studies (Bellander et al., 2015; Polito et al., 2015; Roheger et al., 2020c). Notably, only two studies investigated exact the same model, including baseline performance and cognitive reserve as predictors. Yet, both were conducted by the same research group (López-Higes et al., 2018a,b).

Regarding statistical methods used to calculate prognostic models, our results show that *n* = 4 studies used multiple regression models (Miller et al., 2013; López-Higes et al., 2018a,b; Roheger et al., 2020c), *n* = 3 studies used latent change score models (Schmiedek et al., 2010; Shing et al., 2012; Bellander et al., 2015), and one study used structural equation modeling (Binder et al., 2016). All studies used change scores as dependent variable with one exception – a study by Schmiedek et al. (2010) which used net effect scores.

### Prognostic Factors and Models of Changes in Performance After Multi-Domain CT

Due to the high heterogeneity of the data and the fact that some data was either missing or not clearly reported, it was difficult to detect a specific pattern of prognostic factors of changes after multi-domain CT (see Tables 2, 3 for a detailed overview of the results).

Regarding prognostic factor studies, 13 studies were investigated. Two studies found that lower baseline performance in the trained task predicted improvements in executive functions (Oswald et al., 1996; Gallen et al., 2016). One study showed that lower age predicted improvements in global cognition, memory, and executive functions (Legault et al., 2011), whereas the other study investigating age in these domains did not find significant results (Oswald et al., 1996). Yet, no more patterns could be detected over the prognostic factor studies. *N* = 2 studies did not report the directions of the results at all (Lövdén et al., 2010; Akimoto et al., 2016), *n* = 5 studies only reported the directions of the significant correlations (Oswald et al., 1996; Legault et al., 2011; Kim et al., 2015; Otsuka et al., 2015; Niu et al., 2016), and did not report directions of non-significant results (results of all studies are depicted in more detail in Table 2).

Four models that included age as a prognostic factor [two using a latent change score model (Schmiedek et al., 2010; Shing et al., 2012), two using multiple regression analyses (Nouchi et al., 2019; Roheger et al., 2020c)] did not find a significant relation between age and changes after multi-domain CT in the domains global cognition (Roheger et al., 2020c), memory (Schmiedek et al., 2010; Nouchi et al., 2019; Roheger et al., 2020c), attention (Nouchi et al., 2019; Roheger et al., 2020c), executive function (Schmiedek et al., 2010; Shing et al., 2012; Niu et al., 2016; Roheger et al., 2020c), language (Roheger et al., 2020c), or visuo-spatial abilities (Roheger et al., 2020c). Only Shing et al. (2012) found a negative correlation in the domain memory, indicating younger participants to benefit more from the training. Baseline performance was integrated as a prognostic factor in three prognostic model studies [one using a structural equation model (Binder et al., 2016), two using multiple regression models (Nouchi et al., 2019; Roheger et al., 2020c)], showing contradictory results: while in one study participants with higher baseline performance benefited most in executive functions (Nouchi et al., 2019), results of the two other studies showed that participants with lower baseline performance benefited in this domain (Binder et al., 2016; Roheger et al., 2020c). Only two studies used exact the same prognostic factors in their model (López-Higes et al., 2018a,b), but investigated different outcome domains, therefore not serving as a validation of their results. They found that lower cognitive reserve leads to more benefit in global cognition (López-Higes et al., 2018a), and that lower cognitive reserve leads to more benefit scores in the language domain (López-Higes et al., 2018b).

## Discussion

The aim of the present review was to identify prognostic factors and models for predicting changes after multi-domain CT in healthy older adults. Our main results are that (i) there is a high heterogeneity not only regarding the conducted multi-domain CT, but also regarding the investigated prognostic factors, the investigated outcomes, and the used statistical approaches, and that (ii) there is a poor reporting of prognostic factor and model studies. Further, (iii) investigated predictors include sociodemographic variables (i.e., age, sex, education), neuropsychological performance at study entry in different tasks and domains, further psychological variables (i.e., quality of life, depression), training characteristics (i.e., modality, intensity), genetic variables (i.e., apolipoprotein E4), brain imaging measures, and EEG markers. Age and baseline performance were the most investigated predictors, but results are inconsistent.

The present review shows that most prognostic factor and model studies show strong methodological shortcomings and therefore conclusions are difficult. Several guidelines for the adequate conduction and reporting of prognostic factor and model studies exist (Moons et al., 2009, 2015; Riley et al., 2013; Steyerberg et al., 2013). Yet, none of the included studies used any of the guidelines designed for the reporting of prognostic models and factors; only two studies (Mayas et al., 2014; Nouchi et al., 2019) stated that they used the CONSORT reporting guideline for RCTs (Cuschieri, 2019). The present review underlines the need to use these guidelines in order to generate evidence-based, reproducible and reliable results as it is not possible to generate these from studies without a clear reporting of predictors or statistical analysis used. In the Supplementary Material, we provide the TRIPOD Statement Checklist (Moons et al., 2015) as an example guideline to demonstrate which aspects are important when reporting prognostic research. Specific explanations and examples can be obtained in the original publication and go beyond the scope of the present review. Furthermore, validation of the results of prognostic research is essential as performance in “a validation study is arguably all that matters, and how a model was derived is of little importance if it performs well” (Steyerberg et al., 2013). However, validation is missing in most of the conducted studies. In the field of non-pharmacological—and more specific, cognitive–interventions, it seems important that the perception of prognostic research changes: from being a solely “add-on analysis” of a present study to the own research methodology with its challenges and obstacles that it is. Without this change of perception and a change in the conduction of prognostic research according to the present guidelines, further development in this research field will not be achieved.

However, keeping the limitations named above in mind, some study results should still be discussed. Regarding sociodemographic predictors, one prognostic factor study and one prognostic model study found younger participants to benefit more from multi-domain CT in the domain memory, while four prognostic model studies could not show a significant relationship between age and performance change after multi-domain CT. While these results are conflicting, they are also not in line with the results found in a recent systematic review on prognostic factors of solely memory training on memory outcomes (Roheger et al., 2020a), in which older participants benefited most from the training. Yet, one possible reason might be that multi-domain trainings challenge a larger variety of domains than a single-domain memory training, so that the multi-domain training might be more complex, and effects are harder to achieve due to the lower intensity of training of specific domains. As a result, it may be easier for younger participants to train in a multi-domain training than older participants. So far, results on prognostic factors on single and multi-domain CT performance have been discussed in the context of the magnification and the compensation account (Lövdén et al., 2012). The magnification account is prominent for interpreting the increase in adult age differences after trainings as it suggests that individual and age-related differences in gains from CT can be explained by initial differences in cognitive resources available to acquire, implement, and sharpen effortful cognitive strategies. In adult lifespan samples cognitive abilities and possible gains from mnemonic training seem to decline with age (Verhaeghen and Marcoen, 1996; Rönnlund et al., 2005). The magnification account predicts that group differences will magnify after the training (Lövdén et al., 2012). On the contrary, the compensation account states that individuals who are already functioning at optimal levels have less room for changes in memory training performance. Older participants may then have more room for cognitive improvement as younger adults. Gains from CT should correlate negatively with cognitive abilities and age differences are reduced after training. Both accounts are still under debate regarding the conditions under which they occur (Karbach and Unger, 2014). Yet, a recent methodological assessment of the existing and potential evidence in favor of the compensation account of CT shows that most of the evidence is highly questionable due to the incorrect use of statistics (Smoleń et al., 2018). Smolen and colleagues showed that a negative correlation of pre-test score and training gain occurs naturally when gain (treated as the dependent variable) is the linear function of the independent variable (pre-test). This is a special example of a general statistical artifact called regression to the mean. Therefore, the authors suggest using graphical and structural equation models when investigating prognostic factors of CT gains, which is also emphasized in the previous cited guidelines on prognostic factor and model research. Notably, while we did not find education to be predictive for changes in multi-domain CT, education is also a factor that should be investigated in more detail in further studies on prognostic factors and models of changes after multi-domain CT. Educational attainment moderated training effects on cognitive outcomes in healthy older adults in the recent meta-analysis by Basak et al. (2020), indicating that participants with less formal education benefitted more from CT (Basak et al., 2020).

Regarding possible neuropsychological predictors, results are also inconsistent. In two prognostic factor and also two prognostic model studies, lower neuropsychological performance at study entry predicted gains in the investigated cognitive outcomes, while one other prognostic model study found higher baseline performance to be predictive for training benefit. Yet, as prognostic model studies included different types of possible predictors additionally to neuropsychological performance at study entry, it is not possible to detect why different results were found. For such an analysis, identical prognostic models in different study samples would be needed to draw clearer conclusions. Again, for the explanation of differences in neuropsychological performance at study entry, the magnification and compensation accounts can be consulted (with consideration of the already outlined methodological critique and the awareness that it is still not clear under which circumstances these two accounts occur). Future research about these two explanation approaches and linked statistical methods is needed to draw clearer conclusions.

In the present review, investigated predictors include sociodemographic variables (i.e., age, sex, education), neuropsychological performance at study entry in different tasks and domains, further psychological variables (i.e., quality of life, depression), training characteristics (i.e., modality, intensity), genetic variables (i.e., apolipoprotein E4), brain imaging measures, and EEG markers. Age and baseline cognitive performance were most used as these are standard covariates used in most studies. However, one has to take into account that there are of course several other potential predictors that might have an influence on CT and that should be considered in future studies on prognostic factors and/or models for changes after CT. Examples are psychological variables including intelligence (Lee et al., 2015), personality traits (Hill et al., 2014), or locus of control (Wolinsky et al., 2010), as well as social factors such as isolation and networking (Evans et al., 2018).

In the present review, studies that used structural equation modeling did not find significant effects when investigating age and multi-domain CT gain, and mixed effects when investigating baseline neuropsychological performance and multi-domain CT gain. Therefore, it has to be emphasized again that more research with adequate statistical analysis and reporting is needed to gather robust evidence on this topic. As a consequence of the methodological shortcomings and the heterogeneity of the studies, no further results on prognostic models can be discussed meaningfully.

There are some limitations that have to be taken into account when reading this review. First, as outlined, data was too rare and heterogeneous to perform a meta-analysis (as we registered in our pre-registration). However, important methodological shortcomings regarding the statistics and reporting could be identified, so that suggestions to improve the data quality can be outlined for future research. Second, as only German and English articles were included in the present review, this implies a possible limitation of the present review due to the fact that we might have missed information of articles in other languages. As a more general limitation regarding research on multi-domain trainings, they are in some cases more complex than single-domain trainings and aim at emphasizing complex cognitive interactions by simultaneously engaging either multiple lower-level mental processes (e.g., attention, memory, etc.) or higher-level executive functions (e.g,. inhibition, flexibility of thinking) (Tagliabue et al., 2018). Consequently, the trained domains of the included studies are naturally highly heterogeneous and it is difficult to pinpoint which aspect of the training actually brings benefit. In addition to that, the optimal amount of different trained modalities will likely differ on an individual basis; therefore, a personalized approach to CTs as it is addressed in this review with the aim to identify possible predictors, might lead to more sustained and significant outcomes (Ball et al., 2007). Furthermore, we decided to exclude videogames and brain trainings, that did not specifically state which cognitive domains they target. Therefore, we might have missed some studies that could potentially be included in the systematic review. Yet, we made this decision as an explicit outline of trained domains was a prerequisite to be able to clearly include multi-domain trainings. Future studies should explicitly investigate effects and responsiveness to videogames and brain trainings, but will have to access more detailed information that goes beyond those available in the manuscripts published.

Summarized, this is the first systematic review on prognostic factors and models of multi-domain CT. Prognostic research is of high importance in informing prevention decisions (either directly or as part of prognostic models for an individualized prediction), and also in improving the design of intervention trials and in targeting new interventions to strengthen cognitive function in older adults (Riley et al., 2013). By fostering knowledge in this field, we will not only be able to state that multi-domain CT is effective in strengthening cognition in healthy older adults (Basak et al., 2020) as a group (which is a highly important message due to the aging population and the related risk for cognitive decline and dementia), but who—with which profile of characteristics—benefits from which CT. Yet, the present review showed that prognostic research in multi-domain CT is still at the beginning: even though clear guidelines exist, most studies have statistical shortcomings and/ or are poorly reported, and results are therefore not reliable. Future prognostic research should focus on using registered study protocols, large sample sizes, appropriate statistical methods, and transparent reporting. Sociodemographic variables (i.e., age, sex, and education), neuropsychological test status at study entry in different domains, further psychological variables (i.e., quality of life, depression), training characteristics (i.e., modality, intensity), genetic variables (i.e., apolipoprotein E4), brain imaging measures, and EEG markers may be possible predictors that influence multi-domain CT gains.

## Data Availability Statement

The original contributions presented in the study are included in the article/Supplementary Material, further inquiries can be directed to the corresponding author/s.

## Author Contributions

MR, HLJ, and EK contributed to conception of the study. FK organized the database. MR wrote the first draft of the manuscript. HLJ and AA wrote sections of the manuscript. AA advised statistical analysis. All authors contributed to manuscript revision, read, and approved the submitted version.

## Conflict of Interest

MR has received a grant from the Brandau-Laibach Stiftung, and a grant from the German Ministry of Education and Research. AA has received a grant from the Brandau-Laibach Stiftung. EK has received grants from the German Ministry of Education and Research, ParkinsonFonds Deutschland GmbH, the German Parkinson Society; honoraria from: Oticon GmbH, Hamburg, Germany; Lilly Pharma GmbH, Bad Homburg, Germany; Bernafon AG, Bern, Switzerland; Desitin GmbH, Hamburg, Germany. EK is author of the CT program NEUROvitalis but receives no corresponding honoraria. The remaining authors declare that the research was conducted in the absence of any commercial or financial relationships that could be construed as a potential conflict of interest.
