# Supplementary material for: Prognostic Factors and Models for Changes in Cognitive Performance After Multi-Domain Cognitive Training in Healthy Older Adults: A Systematic Review
Source: Front Hum Neurosci. 2021 Apr 27;15:636355. doi: 10.3389/fnhum.2021.636355 (PMC8110835; doi:10.3389/fnhum.2021.636355)
Supplement: Supplementary file 1 [file Table_1.docx]

Supplementary Material:

*Prognostic factors and models for cognitive performance changes after multi-domain cognitive training in healthy older adults: a systematic review*

Table 1: The PRISMA for Abstracts Checklist

| **TITLE** | **CHECKLIST ITEM** | REPORTED ON PAGE # |
| --- | --- | --- |
| 1. Title: | Identify the report as a systematic review, meta-analysis, or both. | 1 |
| **BACKGROUND** |  |  |
| 2. Objectives: | The research question including components such as participants, interventions, comparators, and outcomes. | p.2, l. 37/38 |
| **METHODS** |  |  |
| 3. Eligibility criteria: | Study and report characteristics used as criteria for inclusion. | p.2, l. 37/38, 40 - 42 |
| 4. Information sources: | Key databases searched and search dates. | p.2, l.39 |
| 5. Risk of bias: | Methods of assessing risk of bias. | p.2, l.43 |
| **RESULTS** |  |  |
| 6. Included studies: | Number and type of included studies and participants and relevant characteristics of studies. | p. 2, l 44 |
| 7. Synthesis of results: | Results for main outcomes (benefits and harms), preferably indicating the number of studies and participants for each. If meta-analysis was done, include summary measures and confidence intervals. | p.2, l. 46/47 |
| 8. Description of the effect: | Direction of the effect (i.e. which group is favoured) and size of the effect in terms meaningful to clinicians and patients. | n.a. |
| **DISCUSSION** |  |  |
| 9. Strengths and Limitations of evidence: | Brief summary of strengths and limitations of evidence (e.g. inconsistency, imprecision, indirectness, or risk of bias, other supporting or conflicting evidence) | p.2,l. 48/49 |
| 10. Interpretation: | General interpretation of the results and important implications | p. 2, l. 50 |
| **OTHER** |  |  |
| 11. Funding: | Primary source of funding for the review. | 1 |
| 12. Registration: | Registration number and registry name. | p. 2, l- 51 |

Table 2: The PRISMA checklist for systematic reviews

| **Section/topic** | **#** | **Checklist item** | **Reported on page #** |
| --- | --- | --- | --- |
| **TITLE** | | |  |
| Title | 1 | Identify the report as a systematic review, meta-analysis, or both. | 1 |
| **ABSTRACT** | | |  |
| Structured summary | 2 | Provide a structured summary including, as applicable: background; objectives; data sources; study eligibility criteria, participants, and interventions; study appraisal and synthesis methods; results; limitations; conclusions and implications of key findings; systematic review registration number. | 2 |
| **INTRODUCTION** | | |  |
| Rationale | 3 | Describe the rationale for the review in the context of what is already known. | 3 |
| Objectives | 4 | Provide an explicit statement of questions being addressed with reference to participants, interventions, comparisons, outcomes, and study design (PICOS). | 3/4 |
| **METHODS** | | |  |
| Protocol and registration | 5 | Indicate if a review protocol exists, if and where it can be accessed (e.g., Web address), and, if available, provide registration information including registration number. | 4 |
| Eligibility criteria | 6 | Specify study characteristics (e.g., PICOS, length of follow-up) and report characteristics (e.g., years considered, language, publication status) used as criteria for eligibility, giving rationale. | 4 |
| Information sources | 7 | Describe all information sources (e.g., databases with dates of coverage, contact with study authors to identify additional studies) in the search and date last searched. | 4 |
| Search | 8 | Present full electronic search strategy for at least one database, including any limits used, such that it could be repeated. | 4 & Suppl. |
| Study selection | 9 | State the process for selecting studies (i.e., screening, eligibility, included in systematic review, and, if applicable, included in the meta-analysis). | 5 |
| Data collection process | 10 | Describe method of data extraction from reports (e.g., piloted forms, independently, in duplicate) and any processes for obtaining and confirming data from investigators. | 5 |
| Data items | 11 | List and define all variables for which data were sought (e.g., PICOS, funding sources) and any assumptions and simplifications made. | 5 |
| Risk of bias in individual studies | 12 | Describe methods used for assessing risk of bias of individual studies (including specification of whether this was done at the study or outcome level), and how this information is to be used in any data synthesis. | 5 |
| Summary measures | 13 | State the principal summary measures (e.g., risk ratio, difference in means). | 5/6 |
| Synthesis of results | 14 | Describe the methods of handling data and combining results of studies, if done, including measures of consistency (e.g., I^2^) for each meta-analysis. | 5/6 |
| **Section/topic** | **#** | **Checklist item** | **Reported on page #** |
| Risk of bias across studies | 15 | Specify any assessment of risk of bias that may affect the cumulative evidence (e.g., publication bias, selective reporting within studies). | 7 |
| Additional analyses | 16 | Describe methods of additional analyses (e.g., sensitivity or subgroup analyses, meta-regression), if done, indicating which were pre-specified. | 7 |
| **RESULTS** |  |  |  |
| Study selection | 17 | Give numbers of studies screened, assessed for eligibility, and included in the review, with reasons for exclusions at each stage, ideally with a flow diagram. | 6 |
| Study characteristics | 18 | For each study, present characteristics for which data were extracted (e.g., study size, PICOS, follow-up period) and provide the citations. | 6 |
| Risk of bias within studies | 19 | Present data on risk of bias of each study and, if available, any outcome level assessment (see item 12). | 7 |
| Results of individual studies | 20 | For all outcomes considered (benefits or harms), present, for each study: (a) simple summary data for each intervention group (b) effect estimates and confidence intervals, ideally with a forest plot. | 6 - 10 |
| Synthesis of results | 21 | Present results of each meta-analysis done, including confidence intervals and measures of consistency. | 6 - 10 |
| Risk of bias across studies | 22 | Present results of any assessment of risk of bias across studies (see Item 15). | 7 |
| Additional analysis | 23 | Give results of additional analyses, if done (e.g., sensitivity or subgroup analyses, meta-regression [see Item 16]). | none |
| **DISCUSSION** |  |  |  |
| Summary of evidence | 24 | Summarize the main findings including the strength of evidence for each main outcome; consider their relevance to key groups (e.g., healthcare providers, users, and policy makers). | 10/11 |
| Limitations | 25 | Discuss limitations at study and outcome level (e.g., risk of bias), and at review-level (e.g., incomplete retrieval of identified research, reporting bias). | 11/12 |
| Conclusions | 26 | Provide a general interpretation of the results in the context of other evidence, and implications for future research. | 12 |
| **FUNDING** |  |  |  |
| Funding | 27 | Describe sources of funding for the systematic review and other support (e.g., supply of data); role of funders for the systematic review. | 1 |

*From:*  Moher D, Liberati A, Tetzlaff J, Altman DG, The PRISMA Group (2009). Preferred Reporting Items for Systematic Reviews and Meta-Analyses: The PRISMA Statement. PLoS Med 6(7): e1000097. doi:10.1371/journal.pmed1000097

Table 3: Prognostic Factor and Model studies to investigate changes after multi-domain Cognitive Training, search string in **MEDLINE**

|  | older adults.mp |
| --- | --- |
|  | elderly.mp |
|  | aged.mp |
|  | healthy aging.mp |
|  | exp Aged/ |
|  | Cognitive Aging/ |
|  | geriatrics.mp |
|  | gerontolog*.mp |
|  | Geriatrics/ |
|  | or/1-9 |
|  | cognitive intervention.mp |
|  | cognitive training.mp |
|  | cognitive based training.mp |
|  | cognitive support.mp |
|  | cognitive enhancement.mp |
|  | cognitive enrichment.mp |
|  | cognitive aid.mp |
|  | cognitive therapy.mp |
|  | cognitive exercise.mp |
|  | cognitive rehabilitation.mp |
|  | cognitive flexibility.mp |
|  | brain train*.mp |
|  | brain fitness.mp |
|  | or/11-23 |
|  | global cogniti*.mp |
|  | memory.mp |
|  | exp Memory/ |
|  | depress*.mp |
|  | Depression/ |
|  | affective disorder.mp |
|  | affective symptoms.mp |
|  | Affective symptoms/ |
|  | mood.mp |
|  | mood disorder.mp |
|  | exp Mood Disorders/ |
|  | quality of life.mp |
|  | life quality.mp |
|  | QoL.mp |
|  | Quality of Life/ |
|  | life satisfaction.mp |
|  | personal satisfaction.mp |
|  | Personal satisfaction/ |
|  | well being.mp |
|  | or/25-43 |
|  | 10 AND 24 AND 44 |
|  | Limit 45 to humans |

Table 4: Prognostic Factor and Model studies to investigate changes after multi-domain Cognitive Training, search string in **CENTRAL**

| #1 | (“older adults”): ti,ab,kw |
| --- | --- |
| #2 | (elderly): ti,ab,kw |
| #3 | (aged): ti,ab,kw |
| #4 | (“healthy aging”): ti,ab,kw |
| #5 | MeSH descriptor: [Aged] explode all trees |
| #6 | MeSH descriptor: [Cognitive Aging] explode all trees |
| #7 | (geriatrics): ti,ab,kw |
| #8 | (gerontol*): ti,ab,kw |
| #9 | MeSH descriptor: [Geratrics] explode all trees |
| #10 | {or #1-#9} |
| #11 | (“cognitive intervention”): ti,ab,kw |
| #12 | (“cognitive training”): ti,ab,kw |
| #13 | (“cognitive based training”): ti,ab,kw |
| #14 | (“cognitive support”): ti,ab,kw |
| #15 | (“cognitive enhancement”): ti,ab,kw |
| #16 | (“cognitive enrichment”): ti,ab,kw |
| #17 | (“cognitive aid”): ti,ab,kw |
| #18 | (“cognitive therapy”): ti,ab,kw |
| #19 | (“cognitive exercise”): ti,ab,kw |
| #20 | (“cognitive rehabilitation”): ti,ab,kw |
| #21 | (“cognitive flexibility”): ti,ab,kw |
| #22 | (“brain train*”): ti,ab,kw |
| #23 | (“brain fitness”): ti,ab,kw |
| #24 | {or #11-#23} |
| #25 | (“global cogniti*”): ti,ab,kw |
| #26 | (memory): ti,ab,kw |
| #27 | MeSH descriptor: [Memory] explode all trees |
| #28 | (depress*): ti,ab,kw |
| #29 | MeSH descriptor: [Depression] explode all trees |
| #30 | (“affective disorder”): ti,ab,kw |
| #31 | (“affective symptoms”): ti,ab,kw |
| #32 | MeSH descriptor: [Affective symptoms] explode all trees |
| #33 | (mood): ti,ab,kw |
| #34 | (“mood disorder”): ti,ab,kw |
| #35 | MeSH descriptor: [Mood Disorders] explode all trees |
| #36 | (“quality of life”): ti,ab,kw |
| #37 | (“life quality”): ti,ab,kw |
| #38 | (QoL): ti,ab,kw |
| #39 | MeSH descriptor: [Quality of Life] explode all trees |
| #40 | (“life satisfaction”): ti,ab,kw |
| #41 | (“personal satisfaction”): ti,ab,kw |
| #42 | MeSH descriptor: [Personal Satisfaction] explode all trees |
| #43 | (“well being”): ti,ab,kw |
| #44 | {or #25-#43} |
| #45 | #10 and #24 and #44 |

Table 5: Prognostic Factor and Model studies to investigate changes after multi-domain Cognitive Training, search string in **Web of Science**

| # 37 | #35 AND #21 AND #7  Refined by: DOCUMENT TYPES: ( ARTICLE )  Indexes=SCI-EXPANDED, SSCI Timespan=All years |
| --- | --- |
| # 36 | #35 AND #21 AND #7  Indexes=SCI-EXPANDED, SSCI Timespan=All years |
| # 35 | #34 OR #33 OR #32 OR #31 OR #30 OR #29 OR #28 OR #27 OR #26 OR #25 OR #24 OR #23 OR #22  Indexes=SCI-EXPANDED, SSCI Timespan=All years |
| # 34 | **TOPIC:** ("well being")  Indexes=SCI-EXPANDED, SSCI Timespan=All years |
| # 33 | **TOPIC:** ("personal satisfaction")  Indexes=SCI-EXPANDED, SSCI Timespan=All years |
| # 32 | **TOPIC:** ("life satisfaction")  Indexes=SCI-EXPANDED, SSCI Timespan=All years |
| # 31 | **TOPIC:** (QoL)  Indexes=SCI-EXPANDED, SSCI Timespan=All years |
| # 30 | **TOPIC:** ("life quality")  Indexes=SCI-EXPANDED, SSCI Timespan=All years |
| # 29 | **TOPIC:** ("quality of life")  Indexes=SCI-EXPANDED, SSCI Timespan=All years |
| # 28 | **TOPIC:** ("mood disorder")  Indexes=SCI-EXPANDED, SSCI Timespan=All years |
| # 27 | **TOPIC:** (mood)  Indexes=SCI-EXPANDED, SSCI Timespan=All years |
| # 26 | **TOPIC:** ("affective symptoms")  Indexes=SCI-EXPANDED, SSCI Timespan=All years |
| # 25 | **TOPIC:** ("affective disorder")  Indexes=SCI-EXPANDED, SSCI Timespan=All years |
| # 24 | **TOPIC:** (depress*)  Indexes=SCI-EXPANDED, SSCI Timespan=All years |
| # 23 | **TOPIC:** (memory)  Indexes=SCI-EXPANDED, SSCI Timespan=All years |
| # 22 | **TOPIC:** ("global cogniti*")  Indexes=SCI-EXPANDED, SSCI Timespan=All years |
| # 21 | #20 OR #19 OR #18 OR #17 OR #16 OR #15 OR #14 OR #13 OR #12 OR #11 OR #10 OR #9 OR #8  Indexes=SCI-EXPANDED, SSCI Timespan=All years |
| # 20 | **TOPIC:** ("brain fitness")  Indexes=SCI-EXPANDED, SSCI Timespan=All years |
| # 19 | **TOPIC:** ("brain train*")  Indexes=SCI-EXPANDED, SSCI Timespan=All years |
| # 18 | **TOPIC:** ("cognitive flexibility")  Indexes=SCI-EXPANDED, SSCI Timespan=All years |
| # 17 | **TOPIC:** ("cognitive rehabilitation")  Indexes=SCI-EXPANDED, SSCI Timespan=All years |
| # 16 | **TOPIC:** ("cognitive exercise")  Indexes=SCI-EXPANDED, SSCI Timespan=All years |
| # 15 | **TOPIC:** ("cognitive therapy")  Indexes=SCI-EXPANDED, SSCI Timespan=All years |
| # 14 | **TOPIC:** ("cognitive aid")  Indexes=SCI-EXPANDED, SSCI Timespan=All years |
| # 13 | **TOPIC:** ("cognitive enrichment")  Indexes=SCI-EXPANDED, SSCI Timespan=All years |
| # 12 | **TOPIC:** ("cognitive enhancement")  Indexes=SCI-EXPANDED, SSCI Timespan=All years |
| # 11 | **TOPIC:** ("cognitive support")  Indexes=SCI-EXPANDED, SSCI Timespan=All years |
| # 10 | **TOPIC:** ("cognitive based training")  Indexes=SCI-EXPANDED, SSCI Timespan=All years |
| # 9 | **TOPIC:** ("cognitive training")  Indexes=SCI-EXPANDED, SSCI Timespan=All years |
| # 8 | **TOPIC:** ("cognitive intervention")  Indexes=SCI-EXPANDED, SSCI Timespan=All years |
| # 7 | #6 OR #5 OR #4 OR #3 OR #2 OR #1  Indexes=SCI-EXPANDED, SSCI Timespan=All years |
| # 6 | **TOPIC:** (gerontolog*)  Indexes=SCI-EXPANDED, SSCI Timespan=All years |
| # 5 | **TOPIC:** (geriatrics)  Indexes=SCI-EXPANDED, SSCI Timespan=All years |
| # 4 | **TOPIC:** ("healthy aging")  Indexes=SCI-EXPANDED, SSCI Timespan=All years |
| # 3 | **TOPIC:** (aged)  Indexes=SCI-EXPANDED, SSCI Timespan=All years |
| # 2 | **TOPIC:** (elderly)  Indexes=SCI-EXPANDED, SSCI Timespan=All years |
| # 1 | **TOPIC:** ("older adults")  Indexes=SCI-EXPANDED, SSCI Timespan=All years |

Table 6: Prognostic Factor and Model studies to investigate changes after multi-domain Cognitive Training, search string in **PsycInfo**

| 1 | older adults.mp. |
| --- | --- |
| 2 | elderly.mp. |
| 3 | aged.mp. |
| 4 | healthy aging.mp. |
| 5 | exp Aging/ |
| 6 | geriatrics.mp. |
| 7 | gerontolog*.mp. |
| 8 | exp Geriatrics/ |
| 9 | exp Gerontology/ |
| 10 | geropsychology/ |
| 11 | or/1-10 |
| 12 | cognitive intervention.mp. |
| 13 | cognitive training.mp. |
| 14 | cognitive based training.mp. |
| 15 | cognitive support.mp. |
| 16 | cognitive enhancement.mp. |
| 17 | cognitive enrichment.mp. |
| 18 | cognitive aid.mp. |
| 19 | cognitive therapy.mp. |
| 20 | cognitive exercise.mp. |
| 21 | cognitive rehabilitation.mp. |
| 22 | exp Cognitive Rehabilitation/ |
| 23 | cognitive flexibility.mp. |
| 24 | exp Cognitive Flexibility/ |
| 25 | brain train*.mp. |
| 26 | exp Brain Training/ |
| 27 | brain fitness.mp. |
| 28 | or/12-27 |
| 29 | global cogniti*.mp. |
| 30 | memory.mp. |
| 31 | exp memory/ |
| 32 | depress*.mp. |
| 33 | exp Major Depression/ |
| 34 | exp "Depression (Emotion)"/ |
| 35 | affective disorder.mp. |
| 36 | affective symptoms.mp. |
| 37 | mood.mp. |
| 38 | mood disorder.mp. |
| 39 | quality of life.mp. |
| 40 | life quality.mp. |
| 41 | QoL.mp. |
| 42 | exp "quality of life"/ |
| 43 | life satisfaction.mp. |
| 44 | exp life satisfaction/ |
| 45 | personal satisfaction.mp. |
| 46 | well being.mp. |
| 47 | exp well being/ |
| 48 | or/29-47 |
| 49 | 11 and 28 and 48 |
| 50 | limit 49 to human |
| 51 | limit 50 to journal article |

Table 7: Risk of Bias Assessment using the QUIPS tool

| Domains and subdomains assessed with the QUIPS tool | Issues to consider according to the QUIPS tool and judgment reasons of the review authors |
| --- | --- |
| **Study Participation** | The domain was rated with high risk, if no inclusion or exclusion criteria were stated, or if more than two subdomains were rated as “high risk”. It was rated as “moderate risk”, if two domains were rated with a “high risk”. |
| - Source of target population | The source population or population of interest is adequately described. |
| - Method used to identify population | The sampling frame and recruitment are adequately described, including methods to identify the sample sufficient to limit potential bias |
| - Recruitment period | Period of recruitment was described. |
| - Place of recruitment | Place of recruitment (setting and geographic location) are adequately described |
| - Inclusion and exclusion criteria | Inclusion and exclusion criteria are adequately described (e.g., including explicit diagnostic criteria or “zero time” description). |
| - Adequate study participation | There is adequate participation in the study by eligible individuals |
| - Baseline characteristics | The baseline study sample (i.e., individuals entering the study) is adequately described for at least the variables age, sex, and education. |
| **Study Attrition** | The domain was rated with high risk, if more than two subdomains were rated as “high risk”. It was rated as “moderate risk”, if two domains were rated with a “high risk” or if either “Reasons for lost to follow-up” or “Outcome and prognostic factor information” was rated with a “high risk”. |
| - Proportion of baseline sample available for analysis | Response rate (i.e., proportion of study sample completing the study and providing outcome data) is adequate. |
| - Attempts to collect information on participants who dropped out | Attempts to collect information on participants who dropped out of the study are described. |
| - Reasons and potential impact of subjects lost to follow-up | Reasons for loss to follow-up are provided. |
| - Outcome and prognostic factor information on those lost to follow-up | Participants lost to follow-up are adequately described, for at least age, sex, and education. |
|  | There are no important differences between key characteristics (age, sex, education) and outcomes in participants who completed the study and those who did not. |
| **Prognostic Factor Measurement** | The domain was rated with high risk, if more than two subdomains were rated as “high risk” or if the subdomain “definition of prognostic factor” was rated as “high risk”. It was rated as “moderate risk”, if two domains were rated with a “high risk”. |
| - Definition of the PF | A clear definition or description of 'PF' is provided. |
| - Valid and Reliable Measurement of PF | Method of PF measurement is adequately valid and reliable to limit misclassification bias. |
|  | Continuous variables are reported or appropriate cut-points (i.e., not data-dependent) are used. |
| - Method and Setting of PF Measurement | The method and setting of measurement of PF is the same for all study participants. |
| - Proportion of data on PF available for analysis | Adequate proportion of the study sample has complete data for PF variable. |
| - Method used for missing data | Appropriate methods of imputation are used for missing 'PF' data. |
| **Outcome Measurement** | The domain was rated with “high risk”, if one subdomain was rated as “high risk”. |
| - Definition of the Outcome | A clear definition of outcome is provided, including duration of follow-up and level and extent of the outcome construct. |
| - Valid and Reliable Measurement of Outcome | The method of outcome measurement used is adequately valid and reliable to limit misclassification bias. |
| - Method and Setting of Outcome Measurement | The method and setting of outcome measurement is the same for all study participants. |
| **Study Confounding** | The domain was rated with “high risk”, if two or more subdomains were rated as “high risk”. The domain was rated with “moderate risk” if one domain was rated with “high risk”. |
| - Important Confounders Measured | Important confounders, including treatments, are measured. |
| - Definition of the confounding factor | Clear definitions of the important confounders measured are provided. |
| - Valid and Reliable Measurement of Confounders | Measurement of all important confounders is adequately valid and reliable (e.g., may include relevant outside sources of information on measurement properties, also characteristics, such as blind measurement and limited reliance on recall). |
| - Method and Setting of Confounding Measurement | The method and setting of confounding measurement are the same for all study participants. |
| - Method used for missing data | Appropriate methods are used if imputation is used for missing confounder data |
| - Appropriate Accounting for Confounding | Important potential confounders are accounted for in the study design or in the analysis. |
| **Statistical Analysis and Reporting** | The domain was rated with “high risk” if the subdomain “Presentation of analytical strategy” was rated as “high risk” or if more than one of the other subdomains was rated with “high risk”. The domain was rated with “moderate risk” if one subdomain (except the first) was rated with “high risk”. |
| - Presentation of analytical strategy | There is sufficient presentation of data to assess the adequacy of the analysis. |
| - Model development strategy | The strategy for model building (i.e., inclusion of variables in the statistical model) is appropriate and is based on a conceptual framework or model. |
|  | The selected statistical model is adequate for the design of the study (e.g. regression model, mixed models). |
| - Reporting of results | There is no selective reporting of results. |

Table 8: Tripod Statement


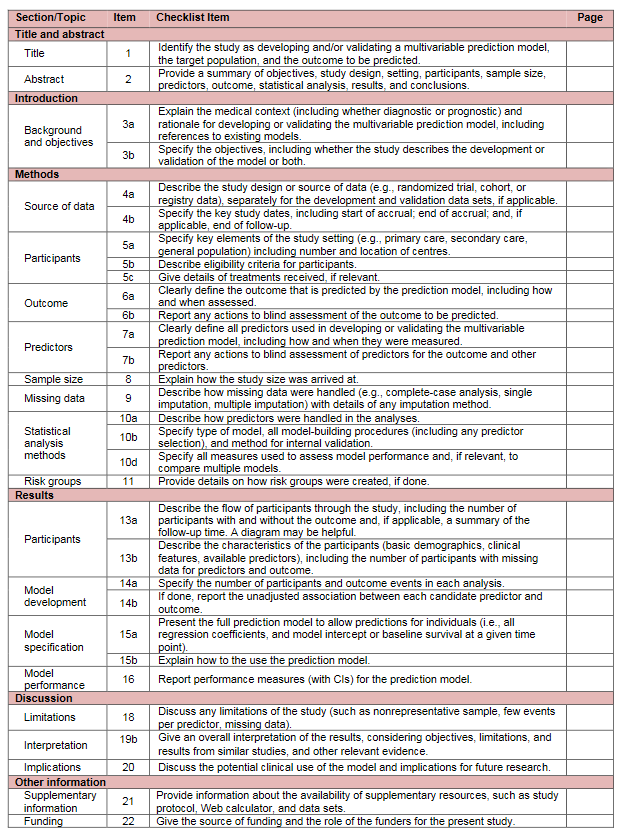


**Source:** Moons, K. G., Altman, D. G., Reitsma, J. B., Ioannidis, J. P., Macaskill, P., Steyerberg, E. W., ... & Collins, G. S. (2015). Transparent Reporting of a multivariable prediction model for Individual Prognosis or Diagnosis (TRIPOD): explanation and elaboration. *Annals of internal medicine*, *162*(1), W1-W73.
